# Draft genome sequence analysis of a multidrug-resistant *Escherichia coli* sequence type 3602 isolated from an equine placentitis lesion in Kentucky, USA

**DOI:** 10.1128/mra.00451-26

**Published:** 2026-10-15

**Authors:** Kirsten E. Scoggin, Susana Bonometti, Justine Elam, Hazem Ramadan, Hossam El-Sheikh Ali

**Affiliations:** 1 Maxwell H. Gluck Equine Research Center, Department of Veterinary Science, Martin-Gatton College of Agriculture, Food, and Environment, University of Kentucky 4530, Lexington, Kentucky, USA; 2 Sumaya Farm, Paris, Kentucky, USA; 3 Hagyard Equine Medical Institute, Lexington, Kentucky, USA; 4 Hygiene and Zoonoses Department, Faculty of Veterinary Medicine, Mansoura University 68779, Mansoura, Egypt; 5 Research Excellence Center, Mansoura National University 68779, Gamasa, Egypt; Fluxus Inc., Sunnyvale, California, USA

**Keywords:** *Escherichia coli*, abortion, equine placentitis, multidrug-resistant (MDR)

## Abstract

We report the draft genome sequence of a multidrug-resistant *Escherichia coli* isolate recovered from an equine placentitis lesion associated with late-term abortion. This genome expands available genomic resources for equine-associated *E. coli* and supports investigation of antimicrobial resistance in equine reproductive disease.

## ANNOUNCEMENT

*Escherichia coli* is an opportunistic Gram-negative pathogen of global importance to humans and animals (1, 2). In horses, *E. coli* is a common cause of ascending placentitis (3). While multidrug-resistant (MDR) *E. coli* strains are described in human infections (4, 5), genomic data for *E. coli* associated with equine fetal loss remain limited. Here, we report the draft genome sequence of an MDR *E. coli* isolate from an equine placentitis lesion.

A swab was collected from the placentitis lesion of a 304-day aborted fetus (mean gestation, 340 days) from a 13-year-old Thoroughbred mare (IACUC #2025-4602). The swab was subcultured onto blood agar at Hagyard Equine Medical Institute and incubated overnight at 37°C. A colony was grown overnight in tryptic soy broth at 37°C. Genomic DNA was extracted using the Qiagen #47014 kit. Genomic DNA purity was assessed using a NanoDrop, yielding A260/A280 and A260/A230 ratios of 1.88 and 2.31, respectively. DNA concentration was measured using a Qubit. DNA integrity was verified by agarose gel electrophoresis by Novogene (Sacramento, CA, USA). Library preparation was performed using the ABclonal RK20208 kit. Sequencing libraries underwent standard quality control at Novogene prior to Illumina sequencing. The sequencing data were of high quality, with 97.64% of bases achieving a Phred quality score of Q30 or higher. Libraries were sequenced on an Illumina NovaSeq X Plus platform, generating 2 × 150 bp paired-end reads. A total of 5,318,053 reads were generated. Reads were quality checked using FastQC (Galaxy 0.12.1) and assembled *de novo* with SPAdes (Galaxy 4.2.0) (6). The genome was annotated with the National Center for Biotechnology Information (NCBI) Prokaryotic Genome Annotation Pipeline (PGAP) v6.10 (7). Genome completeness and contamination were estimated using CheckM2 v1.1.0 (8). Genome analyses were performed using the Center for Genomic Epidemiology pipeline, including multilocus sequence typing (MLST) 2.0, ResFinder 4.7.2, PlasmidFinder 2.1, and VirulenceFinder 2.0 (https://www.genomicepidemiology.org/). Results were validated using ABRicate on the Galaxy server (https://galaxy-main.usegalaxy.org/) with minimum identity and coverage thresholds of 80%. Default parameters were used for all software.

The final assembly comprised 136 scaffolds totaling 5,009,686 bp, with 319× sequencing coverage, an N50 of 144,380 bp, 50.5% guanine-cytosine (GC content), 100% completeness, and 0.15% contamination. The isolate belonged to ST3602 and carried an IncB/O/K/Z replicon, six antimicrobial resistance genes, and 18 virulence-associated genes (Table 1). Antimicrobial susceptibility testing was determined by microdilution using the Sensititre ARIS HiQ, and the obtained minimum inhibitory concentrations were interpreted per the guidelines of the Clinical and Laboratory Standards Institute (9). Susceptibility profiles are shown in Table 1. These ST3602 genomic features highlight emerging MDR *E. coli* in equine reproductive disease and expand the limited genomic resources for equine-associated *E. coli*.

**TABLE 1:** Genomic characteristics, antimicrobial resistance, and virulence-associated genes of *Escherichia coli* strain HEA_MP_H224

Genomic feature	Result
Total reads	5,318,053
Read length	2 × 150 bp
Sequencing coverage	319×
Initial assembly size	5,119,379 bp
Final assembly size	5,009,686 bp
No. of scaffolds (≥500 bp)	136
N_50_	144,380 bp
GC content	50.5%
Genes (total)	4,976
Protein-coding genes	4,710
RNA genes	80
rRNAs	1, 2 (16S, 23S)
Complete rRNAs	1 (16S)
Partial rRNAs	2 (23S)
tRNAs	67
ncRNAs	10
Pseudogenes	186
CRISPR arrays	2
Plasmid replicon	IncB/O/K/Z
Plasmid-associated contig	JBVQSR020000015.1
Antimicrobial resistance genes	*bla*TEM-1B, *sul2*, *tetB*, *dfrA1*, aac(3)-IId, *aadA1*
Antimicrobial susceptibility phenotype^*a*^	Amikacin (≤2 µg/mL, S), ampicillin (>16 µg/mL, R), ceftiofur (0.50 µg/mL, S), chloramphenicol (8 µg/mL, S), ciprofloxacin (≤1 µg/mL, S), doxycycline (>8 µg/mL, R), gentamicin (8 µg/mL, R), imipenem (≤1 µg/mL, S), nitrofurantoin (≤16 µg/mL, S), polymyxin-B (≤1 µg/mL, S), tetracycline (>8 µg/mL, R), ticarcillin-clavulanate (≤8 µg/mL, S), and trimethoprim-sulfamethoxazole (>4 µg/mL, R).
Virulence-associated genes	Adhesion and colonization (*fdeC*, *csgA*, *tia*, *yghJ*, *iha*, *fimH*, *yehA*, *yehB*, *yehC*, *yehD*), invasion (*shiA*), capsule formation (*neuC*), serum resistance (*traT*), tellurite resistance (*terC*), toxin production (*hlyE*, *astA*), and regulatory/accessory (*hha*, *nlpI*).

^*a*^ Minimum inhibitory concentration (MIC) values represent the lowest concentration (µg/mL) of an antibiotic that inhibits growth of the organism tested *in vitro*. Most MIC values are based on veterinarian general use taken from CLSI guidelines. R, resistant; I, intermediate; S, susceptible.

## Data Availability

The whole-genome shotgun project for *Escherichia coli* MDR strain HEA_MP_H224 has been deposited in GenBank under the accession number JBVQSR000000000.2. Raw Illumina sequencing reads have been deposited in the NCBI Sequence Read Archive (SRA) under BioProject PRJNA1426348. All data are associated with BioSample SAMN55864723.
